# Transformed Parenthood in the Face of ALS: A Profound Struggle for Both Ill Parents and Co-parents

**DOI:** 10.1177/23333936251348143

**Published:** 2025-07-12

**Authors:** Nina Malmström, Joakim Öhlén, Stefan Nilsson, Ingela Nygren, Peter M. Andersen, Birgitta Jakobsson Larsson, Anneli Ozanne

**Affiliations:** 1 Institute of Health and Care Sciences, Sahlgrenska Academy, University of Gothenburg, Gothenburg, Sweden; 2 Centre for Person-Centred Care, University of Gothenburg, Gothenburg, Sweden; 3 Palliative Centre at the Sahlgrenska University Hospital Region Västra Götaland, Gothenburg, Sweden; 4 Queen Silvia Children’s Hospital, Sahlgrenska University Hospital, Gothenburg, Sweden; 5 Department of Neurology, Uppsala University Hospital, Uppsala, Sweden; 6 Department of Medical Sciences, Uppsala University, Uppsala, Sweden; 7 Department of Clinical Sciences, Neurosciences, Umeå University, Umeå, Sweden; 8 Department of Public Health and Caring Sciences, Uppsala University, Uppsala, Sweden; 9 Department of Neurology, Sahlgrenska University Hospital, Gothenburg, Sweden

**Keywords:** amyotrophic lateral sclerosis, motor neuron disease, palliative care, parenthood, caregiver, family support, grief, suffering, phenomenological hermeneutics, Sweden

## Abstract

When a parent is diagnosed with a progressive, fatal neurodegenerative disease, such as amyotrophic lateral sclerosis (ALS), it can have major effects on the family’s health. Parenthood itself may also be affected, potentially fueling an urgent need for support from healthcare. Research focusing on this group of parents is nevertheless limited. The aim of this study was to illuminate the meaning of parenthood when a parent has ALS, from the perspective of ill parents and co-parents. An interpretive qualitative study was conducted, using data gathered from interviewing 26 parents (13 ill parents and 13 co-parents) with children living at home in Sweden. Applying a phenomenological hermeneutical analysis, structural analyses depicted the burdensome, complex impact that ALS can have on parenthood, redefining its meaning while forcing parents to face the difficult challenges it brings. The interpreted whole revealed how navigating this transformed parenthood meant a profound struggle, as the parents strived to balance their own emotional pain from grief and worry with remaining stable and supportive for their children. To promote the health of families affected by ALS, more proactive, tailored support is needed within ALS nursing, along with early integration of a palliative approach and attention to the parental perspective.

## Introduction

When a parent is diagnosed with a progressive, fatal neurodegenerative disease, such as amyotrophic lateral sclerosis (ALS), it impacts the entire family (Bergem & Aamotsmo, 2024; Malmström et al., 2024; Sommers-Spijkerman et al., 2022; Testoni et al., 2023). Parents may also face significant challenges related to their parental roles, where professional help may be needed. However, to inform the development of effective support, it is important to further explore the lived experiences and meanings of parenthood in families affected by ALS.

ALS is characterized by its frequently aggressive and inevitable progression, resulting in increasing bodily limitations (Masrori & Van Damme, 2020). This can prevent ill parents from carrying out daily activities and participating in family life (Malmström et al., 2024; Sommers-Spijkerman et al., 2022). By gradually destroying the motor neurons in the brain, brainstem and spinal cord, ALS leads to atrophy and weakness of the skeletal muscles, eventually causing generalized paralysis and impaired breathing, swallowing and speech (Masrori & Van Damme, 2020). The disease can also have a cognitive impact, to varying degrees (Pender et al., 2020). Although the etiology of ALS is heterogeneous, 5% to 10% of patients have a family history (Masrori & Van Damme, 2020). The average survival time from onset is 3 to 5 years and there is currently no effective treatment apart from the gene therapy drug tofersen, for an inherited variant of ALS. The drug riluzole also extends survival to some degree, but care otherwise focuses on nutritional and respiratory support, symptom relief, and palliative care. According to the guidelines from the European Academy of Neurology for the management of ALS, care should be provided by a multidisciplinary team, including a specialist nurse, with an emphasis on coordinated care that addresses the needs of both patients and their families throughout the course of the disease (Van Damme et al., 2024).

Parenthood is a complex task filled with challenges, but when a parent is diagnosed with a life-threatening disease, these may be amplified and transformed (Bergersen et al., 2022). Being a parent in a family marked by ALS may be particularly hard, conceivably meaning a multifaceted burden for both the ill parent and the co-parent. Research indicates that parents with ALS can find it more difficult to accept the diagnosis if they have minor children (Foley et al., 2014, 2016; Ozanne et al., 2013). They may even adjust the role of healthcare in their daily life, depending on their children’s reaction, sometimes even foregoing it to shield them from its impact (Foley et al., 2014). Being the partner of a person with ALS has been highlighted as particularly demanding and sorrowful, evoking feelings of injustice, isolation, and being imprisoned by the proximity of the death (Ozanne et al., 2015). As it also involves being a caregiver and a parent at the same time, the partner may face additional challenges in providing support to both the ill parent and children while simultaneously worrying about their children’s well-being and future (Bergem & Aamotsmo, 2024; Sommers-Spijkerman et al., 2022).

Having a seriously ill parent or experiencing the death of a parent while growing up increases the risk of developing several long-term health issues (Guldin et al., 2015; Kaasbøll et al., 2021; Phillips, 2014). As parents are often children’s primary source of security and support when struggling with stressful and traumatic life events, such as dealing with serious illness in the family (Bergersen et al., 2022), it can be hard to know how best to help the children. Simultaneously, previous findings show how the adolescent children of parents with ALS need increased support from healthcare (Malmström et al., 2023), and such situations may present challenges that urgently require support for the parents themselves. However, research focusing on this group of parents is too limited to develop specific supportive programs. A small, recently published study has explored the experiences of co-parents when the other parent has ALS (Bergem & Aamotsmo, 2024), but to our knowledge, no other previous research focuses solely on parenthood. Experiential knowledge from the perspectives of both ill parents and co-parents is important to inform the development of supportive programs. This is further motivated for the implementation of early integrated palliative care (cf. Abel & Kellehear, 2022; Sawatzky et al., 2016; Sallnow et al., 2022).

To meet the needs of families affected by ALS, it is critical to build on both ill parents’ and co-parents’ own experiences, as related to their life situation and the challenges they face as parents. Deeper insights into these lived experiences are needed to better understand how support might be developed within both ALS nursing and palliative care—an area of growing relevance both internationally and in Sweden. The aim of this study therefore was to illuminate the meaning of parenthood when a parent has ALS, from the perspective of ill parents and co-parents.

## Methods

### Design

This study is part of a larger project aimed at improving support for families living with ALS, with three related studies already published (Malmström et al., 2023; Malmström et al., 2024; Nilsson et al., 2025). By reporting the specific aim pertaining to the meaning of parenthood, the purpose of this study was to contribute to a deeper understanding to inform and strengthen the support developed for families within the larger project. The study employed an interpretive qualitative design guided by a phenomenological hermeneutics, based on Ricœur’s (1976) theory of interpretation and methodologically developed by Lindseth and Norberg (2004, 2022). Phenomenological hermeneutics was chosen because it is suitable for analyzing meanings through interpretation of text, thus enabling a deeper understanding of experiential and contextual phenomena of human life (Öhlén & Friberg, 2023). The approach may also be particularly valuable for capturing sensitive and complex lived experiences. It has previously been applied in studies involving for example minor siblings of children receiving home mechanical ventilation (Israelsson-Skogsberg et al., 2019), as well as both family caregivers of, and persons living with, other neurodegenerative conditions such as Parkinson’s disease and Alzheimer’s disease (Rosengren et al., 2021; Tambunan & Simbolon, 2023), supporting its relevance in the context of ALS as well. Phenomenological hermeneutics was also used in our previous study into meanings for adolescents of living with a parent with ALS (Malmström et al., 2024).

### Healthcare Context

The study was conducted in Sweden, where the healthcare system provides universal care coverage, including social home care services for persons with severe disabilities, regulated by the Health and Medical Services Act (HSL) and the Act concerning Support and Service for Persons with Certain Functional Impairments (LSS). These services range from personal and household support to advanced medical care by nurses and physicians, depending on needs. People, 66 years of age and younger, may be granted 24-hr personal assistance, while fees for other services are based on the individual’s financial situation. Employees may receive sick pay covering up to 80% of their salary, and family caregivers can apply for compensation for up to 100 days (Swedish Health and Medical Services Act (HSL), 1982, p. 763; Swedish Act concerning Support and Service for Persons with Certain Functional Impairments (LSS), 1993, p. 387). Most patients with ALS in Sweden are cared for by a multidisciplinary ALS team at a hospital, with home-based services through municipal, primary and palliative care gradually introduced as the disease progresses (Swedish National Board of Health and Welfare, 2024). A recent registry study showed that 95% of patients with ALS (aged 18–64) in Stockholm lived at home (Strang et al., 2024).

### Sampling and Participants

The participants were recruited via ALS teams at three university hospitals in different parts of Sweden. Recruiting via these teams was a purposive choice to obtain participants with current experience, while involving three different hospitals was strategic to achieve variety. The teams were instructed to invite everyone who met the inclusion and exclusion criteria (Table 1). The ill parents were informed about the study and asked whether they were interested in participating. With their consent, their partners/former partners with whom they had children were contacted and provided with similar information. Twenty-seven parents agreed to participate, none of whom were excluded. However, one ill parent ultimately declined, citing the illness as the hindering factor.

**Table 1.** Inclusion and Exclusion Criteria for Participants.

	Inclusion criteria	Exclusion criteria
Ill parents	• Diagnosed with probable or definite ALS according to the El-Escorial WFN* (Brooks et al., 2000) at least six months earlier • Treated by an ALS team • Has sufficient communication skills to be able to participate in an interview • Has children between the ages of 8–25 who live at home full-time or part-time	• Dementia or severe cognitive impairment • Psychological impact making participation unsuitable according to the treating ALS team
Co-parents	• Partner/former partner of a person with ALS • Has children (between the ages of 8–25 who live at home full-time or part-time) with the person with ALS	• Co-parents who the ill parent did not want to include • Psychological impact making participation unsuitable according to the treating ALS team

* World Federation of Neurology.

In this study, the term “parents” refers to both ill parents and co-parents as a group, focusing on similarities, while “ill parents” and “co-parents” are used to specifically describe each group separately. The term “children” refers to the children as children of their parents, and not in terms of maturity. The term “family” is used and understood as a relational unit shaped by lived experience. Although the meaning and structure of ‘family’ may vary between individuals, this study focuses on biological and/or legal parenthood, while allowing for variation in family constellations, such as cohabiting and separated parents as well as families with adopted children.

### Data Generation

Between December 2020 and April 2022, individual interviews were conducted by two of the authors (NM, AO) and a research assistant. None of the latter were involved in the care of the ill parents. The research team brought together expertise from several disciplines: public health; nursing, including ALS care, palliative care, and pediatric care; and neurology with expertise in ALS care. The team also possessed methodological expertise in narrative interviewing and phenomenological hermeneutics.

To reduce the burden on the already vulnerable group of participants, interviews were conducted on a single occasion, with interviewers remaining attentive and careful not to exceed individual needs or limits. An explorative interview guide, developed without a predefined theoretical framework, was used as support by all three interviewers, allowing for flexibility and inclusion of follow-up questions. The interviews covered both the meaning of parenthood and children’s need for professional support, but this study analyzed only parenthood-related data; findings on children’s support needs have been published separately (Malmström et al., 2023). Two pilot interviews were conducted, resulting in a reduced number of questions; these interviews were later also included in the analysis. The main questions on the meaning of parenthood from the interview guide are presented in Supplemental material 1. The interviews were performed face-to-face or by telephone, depending on the participants’ preference, lasted between 17 and 99 min (median = 47 min), and were audio-recorded and transcribed verbatim.

### Data Analysis and Interpretation

Although most of the research team had both research and clinical experience in ALS and/or palliative care, the main part of the analysis was conducted by the first author, who did not have a background in these fields, but instead in public health—potentially allowing for a more open and explorative approach, less influenced by clinical pre-understandings. However, four of the other authors (JÖ, SN, BJL, AO) were also involved throughout the analytic process via continuous discussions and reflections on emerging interpretations. In the final stages, all authors participated, which contributed to both reflexivity and trustworthiness. The interviews were analyzed using the methodology of Lindseth and Norberg (2004, 2022), following the three interrelated steps of phenomenological hermeneutics: naïve reading, structural analyses and interpreted whole. Through the naïve reading of the interviews, a comprehensive understanding of overall meanings was created, which also resulted in analytical questions to guide the subsequent structural analyses. Initially, parts of the text addressing these topics were thoroughly read through and then divided into separate meaning units. Subsequently, analyses of the data associated with each of the questions were carried out and abstracted into meaning clusters, capturing patterns and variations of meaning in the interview text to inform the final interpretation. To reach an interpreted whole of the investigated phenomenon, the naïve reading and the structural analyses were then woven together and interpreted in relation to each other, but also to the authors’ pre-understandings. The authors returned to the full text, reviewed the findings, and reflected on how the naïve understanding and the structural analyses informed and complemented each other, with the aim of deepening the interpretation and achieving a better understanding of the essential meaning of the lived experience. Here, the use of relevant literature and theoretical perspectives is encouraged to support and enrich the understanding, not by imposing a perspective on the findings, but by allowing the literature to illuminate them and vice versa (Lindseth & Norberg, 2004, 2022). Accordingly, *Sense of Coherence* (Antonovsky, 1987) and *Dual-Process Model of Grief* were applied (Stroebe & Schut, 2010). Table 2 illustrates how the analysis developed through the three interrelated steps: starting with the naïve reading, whose questions guided the structural analyses where meaning clusters were formed, while movement between the parts and the whole throughout the process contributed to reaching the interpreted whole. With the naïve reading and the structural analyses written in the imperfect tense to reflect what the participants shared, the interpreted whole is written in the present tense, to demonstrate how the investigated phenomenon can be understood.

**Table 2.** An Overview of the Results.

The naïve reading			
The parents struggled to maintain parenthood, as ordinary family life had been turned upside down and replaced by an everyday life full of illness and uncertainty. It seemed to be an overwhelming challenge, with the parents grieving everything in life that did not turn out as envisioned while also worrying about how it was affecting the children and how to adequately support them. Additionally, the parents’ narratives included descriptions of being left to deal with this enormous burden, and of grappling with their own difficult feelings while trying to guide the children.			
Structural analysis 1: Dialectic between vulnerability and parental strength	Structural analysis 2: Navigating a transformed parenthood		
How did the parents share their experiences of parenthood?	What did the changed parenthood mean?	What did the worry mean?	What did the grief mean?
A spectrum from hopelessness to renewed perspective	Changing parental roles Maintaining a sense of normalcy for the children Struggling in communication with the children Seeking parental support in the social network	Worrying about not being able to support the children Worrying about the children’s increased responsibility Worrying about the family’s uncertain future Worrying about the impact of home care	Grieving a lost everyday life with the children Grieving a lost future with the children
Interpreted whole			
The meaning of parenthood while living with ALS can be understood as a profound struggle to navigate a transformed parenthood for both the ill parents and the co-parents. Comprehensibility, manageability, and meaningfulness are constantly tested as the parents try to balance their overwhelming emotional pain in grief with their efforts to remain a stable, supportive presence for the children.			

### Ethical Considerations

The study was performed in adherence with the principles of the Declaration of Helsinki (WMA, 2013). Ethical approval was obtained from the Swedish Ethics Review Authority (Dnr 2019-03861). Verbal and written informed consents were provided by all participants prior to participation.

## Results

The final sample (*N* = 26) included 12 men, and 14 women aged between 35 and 66 (mean = 53 (*sd* = 8.2), median = 54). Half of the participants were ill parents, while the other half were co-parents, and either current or former partners. All participating parents had at least one child between the ages of 8 and 25 living at home, in accordance with the inclusion criteria. Some also had children outside of this age range, with the youngest child being 2 years old and the oldest 35. Time from diagnosis to interview varied from <1 to 10 years, with the largest group (4 out of 13 ill parents) in the 1 to 2 years interval. To assess the functional disability of the ill parents, the Amyotrophic Lateral Sclerosis Functional Assessment Scale-Revised (ALSFRS-R) was used (Cedarbaum et al., 1999). The total score ranges from 0 to 48, with a higher score indicating better preserved physical function. The total score varied between 9 and 45, with a mean of 31.2 (*sd* = 12.5, median = 37). Nine ill parents had sporadic ALS, one had familial ALS, one was unaware of the type, and data for this question were missing for two parents.

Through the analytical questions generated by the naïve reading, two meaning structures were constructed from the narratives about parenthood when a parent has ALS: *dialectic between vulnerability and parental strength* and *navigating a transformed parenthood*. See Table 2 for an overview of the results.

### Structural Analysis 1: Dialectic Between Vulnerability and Parental Strength

#### How Did the Parents Share Their Experiences of Parenthood?

##### A Spectrum From Hopelessness to Renewed Perspective

When discussing parenthood in this predicament of illness, the parents related experiences which ranged from feeling hopeless to having a renewed perspective. For some, it was total chaos, with no hope of alleviating the family’s pain. They tried facing the situation and were forced to either “grit their teeth or break down.” Attempts to handle it included making the most of the remaining time and finding positives, such as in unifying the family, creating stronger relationships with the children, or valuing their time together more. Several parents highlighted gaining a new perspective on life, as illustrated in the quote below:

Interviewer: “How do you see your parenthood now that you have had the disease for a while?”

Ill parent 1: “I think I’m a good parent. (. . .) My focus has been. . . to dare. Yes, to choose life instead of choosing all the negative parts, of not choosing life. I’ve really chosen life, and life together with my children. . . Yes, my main focus has always been on what is important in life and not on what isn’t important at all. And the most important thing is, of course, the children. After all, there has been a lot of focus on them having a good time. . .//. . .But. . . I’ve probably also focused on myself having a good time with them.”

Within the spectrum from hopelessness to renewed perspective, vulnerability and parental strength were reflected as being dialectically related, where these two opposites seemed to be tightly intertwined rather than implying the absence of the other. While strength was manifested in the parents’ efforts to protect their children, vulnerability was evident in their emotional distress. They struggled to navigate feelings of grief, worry and powerlessness, and they endeavored to be strong for their children by maintaining stability and structure. This balancing act presented challenges to their parenthood and triggered feelings of inadequacy. Here, vulnerability was also reflected in the parents’ experience of failing their children, while their strength drove them to continue trying to be the parent they wanted to be. Likewise, vulnerability and strength were depicted in their oscillation between avoidance and confrontation of the future, with the reluctance to imagine life without each other weighed against the practical need to plan for it.

### Structural Analysis 2: Navigating a Transformed Parenthood

The parents’ narratives of navigating a transformed parenthood related to their experiences of living in this changed parenthood while struggling with constant worry and profound grief.

#### What Did the Changed Parenthood Mean?

The parents’ narratives revealed the profound impact of the illness on their roles and relationships, demonstrating a changed parenthood. Driven to protect their children, they sought strategies to maintain normalcy for the children, communicate appropriately, and seek help from their social network, while calling for improved parental support.

##### Changing Parental Roles

As parental roles increasingly changed because of the impact of the illness on family life, the parents found it challenging to be the parent they wanted to be to their children. Limited emotional strength, time, or bodily ability were often obstacles to fulfilling their parental responsibilities, leading to feelings of inadequacy.

The ill parents struggled with losing their former parental role, describing themselves as “becoming a burden on the rest of the family.” This triggered feelings of guilt and shame about being a hindrance to the family but was also a driver to continue participating in everyday life. Several ill parents described having more time for their children and becoming more present in interactions. Others chose to withdraw from family and social activities because their presence, which required planning, logistics and assistance, became inconvenient for the family. They also worried that their distinctive appearance might embarrass the children, with some sharing painful experiences of their younger children negatively comparing them to friends’ parents.

The co-parents faced a growing burden due to the illness, describing the predicament as “having to be a double parent.” As they became increasingly preoccupied with household responsibilities and caregiving duties, they worried about the lack of time for the children. One co-parent shared: “I can’t give my child the same amount of time. That’s probably the word I’m looking for: time” (Co-parent 1).

The parents prioritized differently in their parental roles and shifted focus “from everyday concerns to things that really mattered.” Especially ill parents with fatigue were forced to choose what to spend energy on. This, combined with the desire for a harmonious final period, could lead them to avoid conflicts with the children. In contrast, co-parents experienced a heavier load, with the children increasingly turning to them to vent their growing frustration, rather than to the ill parents. Simultaneously, the co-parents strove to strengthen their bond with their children in “creating a ‘we’ to remain strong together in the face of future challenges.”

Professional support was believed to help parents come to terms with possible new parental roles. However, co-parents mentioned there were challenges in seeking support if the ill parent refused to discuss the disease, while they themselves felt excluded and in need of individual attention. Limited energy and time also made it difficult to fit in additional appointments. Parents whose children had been born after the diagnosis emphasized the need for healthcare to discuss future pregnancies and parenthood with patients of childbearing age, both individually and as a couple.

##### Maintaining a Sense of Normalcy for the Children

Maintaining a certain structure for the children and identifying ways for the family to live as normally as possible was considered important. As one co-parent described it: “We’ve talked about it a lot, my partner and I. (. . .) To try to maintain. . . as normal a life as possible, for the children’s sake too” (Co-parent 2). This meant ensuring that the children could keep their routines with school, friends, and leisure activities, but it also involved finding and adapting opportunities for the ill parents to continue participating in family life. Several parents described how they strove to have fun and “create meaningful memories” together as a family, while emphasizing the importance of children and co-parents getting a break from the illness. In addition to more practical help, support for parents to identify strategies to facilitate parenthood was requested.

##### Struggling to Communicate With the Children

The parents said it was difficult to know how to communicate with their children about the disease. Some tried to shield their children by withholding information, such as only mentioning that the parent had a condition that weakened the muscles, but without naming the diagnosis or talking about it as incurable and fatal. This seemed to be more common in families with younger children. Others believed that increased knowledge could reduce the children’s worries and therefore chose to be as honest and transparent as possible. The parents also tried to encourage the children to come directly to them instead of searching for answers on the Internet. Although some parents described their children as open, several struggled to reach them. Additionally, ill parents in particular shared how they endeavored to positively influence their children regarding the possibility of new effective treatments.

The approach taken depended on the children’s age, maturity, and the disease stage. While most parents agreed with their partner/former partner on how to include the children, some disagreed on the timing of revealing the diagnosis. In these families, ill parents preferred to wait, while co-parents pushed ahead. Waiting could be a strategy to protect the children, but also to protect the parents while they needed time to first process it themselves, or to hold on to the vision of still “being healthy”.

The reason I didn’t tell the children about it until later, it was simply because I didn’t know what to say. I didn’t even know what this was myself. It didn’t exist in my world. And I tried to find out as much as I could, so that I at least could give them an answer. Because the worst thing I knew was. . . if they ask me something and I just say: ‘I don’t know’, then they just get more worried. – Ill parent 2

Parents expressed a need for tools and strategies to appropriately communicate these topics, in particular when preparing to disclose the diagnosis, although help with communication could also be necessary at other stages.

##### Seeking Parental Support in Their Social Network

Supportive relationships and a good social network were described as important to maintaining parenthood. This could include getting help with everyday chores, daily parenting or caregiving duties, but also meant reducing loneliness. One family had chosen to involve friends and relatives and wrote them a letter to explain how they wanted to be treated, which resulted in appreciative reactions and helpful support. Others avoided discussing the situation to prevent being labeled as victims or being treated differently. Poor knowledge about the disease was also perceived as frustrating. Additionally, social support could be problematic if the wider family lived far away:

We have support, we do. My parents and my partner’s parents, they’re still quite young, but it’s also. . . people have their lives. My siblings also work and live quite far away. So, it’s also about the practical things. Everyone is supportive, of course, but they can’t be here day and night. (. . .) We have support, but not constantly. They live their own lives. – Co-parent 3

Finding support within the family was seen to strengthen relationships with the children. Some also felt it brought siblings closer, giving the parents comfort in knowing that they would probably be there for each other in the future.

Support from the school played a significant role for several parents. While emphasizing the importance of respecting the children’s opinions about school involvement, some also described challenges in balancing their own willingness to inform the school and their children’s reluctance to allow this. Although the experiences of support groups varied, many appreciated sharing experiences and finding support from other parents in similar situations.

#### What Did the Worry Mean?

The meaning of parenthood in this illness-ridden life situation seemed strongly associated with living under constant worry. The parents worried about not being able to adequately support their children and the increasing responsibility placed on the children and the co-parents, but also about the family’s uncertain future and the impact on the family during home care.

##### Worrying About Not Being Able to Support the Children

The parents struggled with concerns about what having a life-threateningly ill parent meant for their children. They worried about not being able to support them adequately, potentially harming their well-being and development. Even when children coped well, strong emotional reactions like anger, anxiety, and sadness were difficult to handle. Facing these reactions seemed particularly difficult for parents with impaired speech function:

My child has been extremely angry. (. . .) My child is difficult to handle, it’s like a minefield. If you say the slightest thing in the wrong way, my child gets very angry. And it’s extra difficult for me as I no longer have the same power or nuances in my voice. – Ill parent 3

The parents expressed difficulties in assessing whether the children’s mood swings were a consequence of the family’s life situation, or a normal part of adolescence. Simultaneously, they worried that their children were keeping burdensome feelings to themselves.

The parents worried about their children constantly stressing about something bad happening, either to the ill parent or the co-parent, and it often triggered sleep difficulties and catastrophizing in the children. The fear that the ill parent would fall, choke or die could also manifest itself in a reluctance to leave the ill parent alone or to share a meal together. Although open communication seemed helpful, the parents still struggled with how to support their children and described frustration in not being able to calm them.

While parental support was considered to potentially help the parents guide their children, most had received little or no professional support. Although some did not feel the need for it, the importance of healthcare addressing issues related to parenthood, especially in the context of diagnosis and rapid progress, was still emphasized. Several parents requested a contact person for parental support, stressing that it would enable them to invest their time and energy in their parenthood, instead of spending it trying to navigate the right path themselves.

##### Worrying About the Children’s Increased Responsibility

The parents’ limited bodily function or lack of time meant that children had to help with household chores to a greater extent than before. This increased responsibility for cleaning, washing, cooking, grocery shopping, looking after siblings, etc. varied depending on the condition of the ill parent and the age of the children. Some children cared for the ill parent and emotionally supported both parents and siblings. Even though the children wanted to help, the parents worried about them shouldering too much responsibility. Co-parents thought “it forced them to grow up faster,” while ill parents described feelings of guilt about preventing their children from continuing their own lives.

The parents struggled with worry over their children’s increased responsibility but also found ways to alleviate it. Some co-parents “kept an eye on the children to ensure they did not take on too much,” while others emphasized the importance of helping each other within the family. Holding onto the belief that contributing could benefit children’s well-being seemed common among the parents, although the ill parents were more likely to highlight the positive aspects, such as increased maturity and independence. Witnessing their children’s personal growth provided reassurance that they would thrive, even without their ill parents.

##### Worrying About the Family’s Uncertain Future

The parents worried about the uncertainty surrounding the disease and the future, and its consequences for the family, especially the children. They struggled with not knowing how much time they had left together or what lay ahead. This uncertainty gave rise to frustration and prevented the parents from providing proper answers to their children’s questions:

I think it’s difficult because. . . in one way, if you are worried, it’s always good to receive information. The problem with this diagnosis. . . is that there’s almost no information. (. . .) It’ll be incredibly difficult for me to. . . say anything to my children, because. . .. (. . .) What is the correct information to give? – Ill parent 4

There was great worry about how long the co-parents would endure, especially during life-sustaining tracheostomy treatment, where they felt trapped in an endless continuum. Co-parents also worried about becoming ill themselves, since the circumstances left no capacity for such an event. Ill parents worried about losing speech and gradually fading, both physically and communicatively, from their children’s lives. The financial and practical impact of the illness, along with impending death, also triggered feelings of guilt in the parents about not being able to support their children’s future needs.

It was common for parents to avoid thinking about what lay ahead and to shield the children from these worries, yet planning together with the partner/former partner was crucial to secure the future. It also provided a sense of control, both by enabling ill parents to remain involved despite limitations and by preparing for life without them. Nevertheless, this process was a major source of stress, especially for parents with younger children:

I felt a lot of stress to get things ready. (. . .) I had a great need to look for a new. . . parental figure, among my friends. (. . .) I really had a great need to, sort of. . . to figure out the future for my child and plan for my child to have a good life. – Ill parent 1

Writing letters, recording voice messages as future legacies, and documenting plans for the children to follow when communication was limited were examples of things the ill parents did in order to continue being a part of the children’s lives. Strategies, such as making a will or reviewing other financial aspects, adapting the house or moving to a new one, were also implemented. Some co-parents were stressed because the ill parents insisted on living at home and sometimes refused home care services. They struggled to discuss home care or respite accommodation, while ill parents worried that nursing homes might be unsuitable and limit their presence in their children’s lives.

The risk of transmitting the disease to their children added to the parents’ uncertainty, and they described different ways of relating to it. Some chose not to discuss it at all, while others had talked with their children about it but kept the information limited to avoid causing fear. Most ill parents had chosen to test whether they had a hereditary variant of ALS, but there were those who deliberately avoided it. One ill parent with familial ALS tried to instill hope in the children by emphasizing that “research was progressing, and a cure would probably be discovered.”

##### Worrying About the Impact of Home Care

Parents feared dependency on home care, worrying about how bringing strangers into their home might affect their children’s comfort and sense of safety, even though this support was valued in maintaining their parental role. Some children were used to the presence of personal assistants and sometimes had good relationships with them. However, it was still challenging, especially with new employees without appropriate training. Witnessing the children having to demonstrate procedures or even staying home from school because no assistant showed up was difficult. Unpleasant situations had also occurred, where both parents and children felt threatened by assistants. Hence, the importance of ensuring potential new employees’ qualifications to work with children and adolescents was emphasized.

In families where older children chose to become their parents’ personal assistants, the ill parents valued their time together but still worried about preventing the children from following the path in life they really wanted. For that reason, there were those who only allowed their children to work part-time as assistants.

#### What Did the Grief Mean?

The parents’ narratives were marked by a profound sense of grief whereby they both mourned the loss of the life they had once lived with their children and families, but also the loss of the future life they had envisioned together.

##### Grieving a Lost Everyday Life With the Children

The parents mourned the loss of the family’s former everyday life and how they increasingly lost control due to the disease. They tried to be prepared while also struggling to “race against time.” Losing the power to decide over their own life triggered feelings of vulnerability. The ill parents also grieved about not being able to participate in their children’s lives as before, as one of them described it: “I don’t have to look that far ahead to understand that what I could do before with my children, I can no longer do. And it feels terrible, both for myself and for my children” (Ill parent 2). The co-parents mourned loss of time with their children, expressing sadness about no longer having the same opportunity to do things together spontaneously. They described loneliness and grief in their changed parenthood, and a feeling that they had simply become “a caregiver.” This sense of loneliness was particularly palpable when the ill parent could no longer speak, as it prevented the co-parent from discussing everyday child-related things with them. However, shifting focus from what the family could no longer do together to what was still possible seemed to be an attempt to deal with the grief over losing their former life:

It’s important to try to focus on what you can still do and adjust accordingly, and not be too fixated on what you can’t do. Which of course is a lot of things, and which is also a part of the grief, but it’s also a grieving process that. . . you just must go through. Everyone’s life changes. – Co-parent 4

##### Grieving a Lost Future With Their Children

Although the parents tried “not to let their thoughts run away with them but to be in the here and now,” there was still a strong sense of grief over the loss of the future. Aware that they were no longer living on the same terms, they mourned the loss of their shared plans and shattered dreams. This grief was intertwined with worrying about the future, and particularly about how the absence of the ill parent would affect the children and their well-being.

Not being able to share their parenthood in the future brought sadness, with some co-parents describing “feelings of panic about soon being left alone with the children.” The ill parents despaired about not being able to witness their children growing up and no longer being a part of their lives. They were sad about not being able to share milestone events in their children’s lives, such as graduations, weddings, or becoming parents themselves. Additionally, co-parents described grief about having to witness their child growing up without their ill parent:

It hurts a little, because as a parent you kind of want your child to have as normal life as possible and we tried as much as we could so that our child would not suffer, but some things you just can’t avoid. – Co-parent 5

Amid all the grief, some parents with older children felt grateful that their children had reached an age where they were no longer dependent on them, which also created a sense of relief. The ill parents found comfort in having shared many good years and cherished memories together.

### Interpreted Whole

When ALS affects a family, parenthood is marked by a complex grief, with one parent soon to die while the other is left alone with the children to continue the life originally intended for them together. The parents experience a range of emotions, from hopelessness to a renewed perspective, also revealing a dialectic between vulnerability and parental strength. Although the parents’ roles gradually change, they strive to maintain a sense of normalcy for their children, communicating with them appropriately and seeking parental support from their social network. Without knowing how to adequately support their children, parents worry about how the illness will affect their children in terms of having to take on increased responsibility, uncertainty around the family’s future, and the impact of home care. These worries are compounded by the grief the parents experience from losing everyday life together, as well as a shared future with their family.

While trying to navigate their transformed parenthood, the parents struggle to understand and handle the situation, seeking to make their remaining time as meaningful as possible. For this reason, *Sense of Coherence (SOC)*, centered on *comprehensibility*, *manageability*, and *meaningfulness*, was chosen to deepen the interpretation of the results (Antonovsky, 1987). Although SOC is shaped by previous experiences, environment and strategies, it can be strengthened and weakened by factors like support, resources, stress, and trauma. The parents in this study grapple with comprehending their situation and trying to help their children understand, albeit hampered in this by the disease’s uncertainty. Simultaneously, they struggle to identify both internal and external resources which can somehow help them handle the unmanageable situation. Meaningfulness is often described as the most important component of SOC. However, the parents face an existence that feels anything but meaningful. Nevertheless, parenthood itself seems to give them a sense of meaning by driving them to endure, for the children. This multifaceted meaningfulness appears to help them find the strength to fight, cherish moments together, and create lasting family memories. The way ALS progresses means there is great fluctuation in comprehensibility, manageability and meaningfulness for the parents because they constantly face new difficulties as the ill parent deteriorates. Here, their ability to adapt and mobilize parental strength to meet these challenges may be reflected in their SOC, thus creating some stability and structure for the children.

Comprehensibility, manageability and meaningfulness may fluctuate (Antonovsky, 1987) due to entering a new phase of the disease, the weight of the co-parent’s burden or the children’s reactions, but also due to the parents’ own grief. Guided by the Dual-Process Model of Grief, the grieving process can be understood as an ongoing balancing act between a *loss-oriented* and a *restoration-oriented process* (Stroebe & Schut, 2010). The dynamic movement between processing difficult emotions associated with the loss and handling practical or social demands that follow is suggested as necessary. However, since these two processes often represent conflicting needs, achieving balance can be challenging, as reflected in the parents’ dialectic between vulnerability and strength. While vulnerability manifests in their struggle with overwhelming feelings, strength emerges in their deep-rooted drive to protect their children from further harm. This internal conflict to suppress emotional pain and maintain manageability may hinder them from fully engaging in their grief, which is a vital part of the healing process (Stroebe & Schut, 2010).

The dialectic between vulnerability and strength challenges parenthood—a challenge reflected in the parents’ fear of failing their children and their determination to provide a stable, supportive presence. In grief (Stroebe & Schut, 2010), the parents move between trying to process the emotional pain of the impending loss or of what their children must go through, and handling the changes it entails. The parents grieve because they feel unable to meet their ideal and lose their parental responsibilities. Both the ill parent and co-parent strive to maintain normalcy for their children while working to accept their limitations. It forces them to redefine their idea of what it means to be “a good parent” and challenges their manageability as they must identify new strategies for interacting with and supporting the children.

Another aspect of the parents’ manageability is reflected in their avoiding confronting the future. They vacillate between protecting themselves and their children from the impending loss and taking responsibility for what will remain. Planning for the future is an obvious part of the restoration-oriented process, but in the reluctance to even think about the time ahead, it overlaps with the loss-orientation (cf. Stroebe & Schut, 2010). For parents, enduring life from day to day is a way of temporarily easing their grief, and something they must balance with facing uncertainty and planning for the time ahead. In addition, their sense of powerlessness regarding this uncertain future comes through in their frustration at not getting answers about what to expect, which further prevents them from maintaining a steady parental role.

From the perspectives of both living with ALS and being a co-parent, the meaning of parenthood can thus be understood as a profound struggle to navigate a transformed parenthood. Comprehensibility, manageability, and meaningfulness are constantly tested as the parents try to balance their overwhelming emotional pain in grief with their efforts to remain a stable, supportive presence for the children.

## Discussion

This study illuminates the burdensome and complex impact of ALS on parents, redefining parenthood as they face emotional pain, practical burden, and bodily limitations that alter their parental role. Ill parents, who felt increasingly helpless, transitioned from being active parents to being dependent on their children, while co-parents juggled caregiving and little time for their children with their own grief. Both ill parents and co-parents struggled to understand, handle and find meaning alongside the rapid progression of the disease, disrupting any sense of consistent control, which left them feeling lost in their parental roles.

Since a strong SOC, that is, high comprehensibility, manageability and meaningfulness, is described as implying a greater ability to handle demanding and stressful life events (Antonovsky, 1987), healthcare support aimed at strengthening this can be valuable. To help parents navigate their illness-ridden and transformed parenthood, the nursing practice *Involvement in the light and in the dark*, could therefore be relevant (Andershed & Ternestedt, 2001, 2020). Developed primarily on the basis of Antonovsky’s (1987) theory, it was originally intended for supporting family members and their involvement in the care of a dying person but may also be applied to the context of this study, with some adjustment. According to the practice, involvement can move between *involvement in the light* and *involvement in the dark*. In relation to our context, parents who feel knowledgeable and supported by healthcare are more likely to experience involvement in the light—which is meaningful involvement. This strengthens their SOC and ability to be the engaged parent they want to be. In contrast, insufficient knowledge and support can lead to involvement in the dark and a weakened SOC, triggering emotional isolation, helplessness and difficulty in maintaining a stable parental presence.

Involvement may fluctuate depending on circumstances, support and resources, and to understand its movement between lightness and darkness, three central components are presented: *to know, to do* and *to be* (Andershed & Ternestedt, 2001, 2020). *To know* is a prerequisite for involvement and linked to comprehensibility, where continuous inclusion in communication with healthcare is crucial. This can strengthen parents’ own comprehensibility, but also their manageability regarding communicating with the children. Additionally, appropriate parental support can help parents understand their children’s feelings and reactions, something our study suggests is in demand. This is reinforced by previous findings showing how parents in families affected by ALS assess their children’s psychosocial health as better than the children themselves, which may indicate a lack of awareness of their actual emotional struggles (Nilsson et al., 2025). *To do* is an action-oriented form of involvement, linked to manageability, and relates to healthcare equipping family members with resources to support the ill person. In our context, parental support, including emotional and practical help, is crucial for maintaining stability for the children. *To be* refers to emotional involvement, deeply tied to meaningfulness, and can also be linked to parenthood as reflected in the results, underscoring the need for a holistic healthcare approach to support parents’ presence in their children’s lives. Ill parents may need support to find meaningful ways to stay engaged in parenthood and family life, while co-parents’ may require help in easing their caregiving burden to focus on parenting and their children’s needs, also confirmed by a previous study (Bergem & Aamotsmo, 2024). A modified framework can provide a practical structure to strengthen parents’ SOC in the pursuit of parental involvement in the light (Andershed & Ternestedt, 2001, 2020).

Previous research highlights communication as crucial in promoting family well-being when a parent has ALS (Sommers-Spijkerman et al., 2022). Simultaneously, our results show how the parents experienced lack of resources to communicate with the children, diminishing their perceived manageability. They were unsure of what and how to tell, while also being worried about having to leave their children’s questions unanswered, which triggered feelings of inadequacy as a parent. Furthermore, differences in perceived communication style within families living with ALS have been observed previously, with parents experiencing it as open while children experience it as closed, and sometimes feel excluded from important conversations (Sommers-Spijkerman et al., 2024). With research suggesting that parents struggle more to accept an ALS diagnosis if they have minor children (Foley et al., 2014, 2016; Ozanne et al., 2013), the parents in this study also seemed more likely to try to protect their children by being silent, if they were younger. However, the importance of involving children in communication when having a life-threateningly ill parent has been emphasized (Dalton et al., 2019). This may possibly be even more acute in ALS, with an often obvious loss of function in a short time and significant caregiving responsibilities sometimes placed on the children (Kavanaugh et al., 2020; Malmström et al., 2024; Sommers-Spijkerman et al., 2022). As children are naturally observant, they may quickly notice signs of deterioration, which, without knowledge, can lead to anxiety and misinterpretations of the situation. Timely and appropriate communication can reduce these risks and improve family cohesion and health (Dalton et al., 2019; Sommers-Spijkerman et al., 2022). Additionally, the adolescent children of parents with ALS express a need to be involved and to understand the disease and its development (Malmström et al., 2023; Malmström et al., 2024). Here, support from healthcare can be crucial in helping parents handle this communication.

Strengthening parental SOC is important, but focusing solely on its components risks neglecting emotional processing. Balancing grief processing with practical adaptation might be the key to the healing process (Fiore, 2021; Stroebe & Schut, 2010). Although the parents oscillated between confronting and distancing themselves from their grief, they needed to suppress emotional pain to endure it. The term *enduring* is central to the *Theory of Suffering*, also rooted in nursing, and is described as distinct from *emotional suffering* by reflecting how people must temporarily separate themselves from their suffering in order to handle it (Morse, 2001). Similarly, a previous study confirms our finding by showing how co-parents push aside their own difficult feelings to instead focus on positive moments in everyday life (Bergem & Aamotsmo, 2024), presumably as a strategy to avoid becoming overwhelmed. However, the healing process also requires emotional pain to be confronted (cf. Stroebe & Schut, 2010). Supporting the parents to achieve balance in this regard may therefore be crucial, allowing them to set aside their grief and suffering to maintain parenthood, while giving them space to process their emotional pain at their own pace. Andershed and Ternestedt (2001, 2020) may help in understanding how support can affect the parents’ ability to navigate between these conflicting states. The aforementioned theoretical perspectives can also be united through SOC by highlighting comprehensibility, manageability, and meaningfulness as central when facing challenging and difficult life events (Antonovsky, 1987). Altogether, it may contribute to a beneficial holistic approach for developing support to guide parents from a parenthood in the shadow of ALS toward parental involvement in the light—with potential relevance for nursing practice.

The guidelines from the European Academy of Neurology emphasize the inclusion of a specialist nurse in the multiprofessional ALS care team, recognizing the central role of nursing in coordinating care and addressing the needs of both patients and their families (Malmström et al., 2024). With their presence in both hospital and home-based care settings, nurses are well positioned to identify challenges early and provide multidimensional support to families living with ALS (Beyermann et al., 2023). Palliative care is another key component of the multidisciplinary ALS care according to the European Academy of Neurology (Van Damme et al., 2024), with previous research showing that its early integration can improve quality of life and care planning for the whole family but also ease the burden on the patient’s family members (Fahrner-Scott et al., 2022). Thus, multiprofessional ALS team care should incorporate a palliative approach in their practice (cf. Sawatzky et al., 2016). A palliative approach has been found to be feasible and appreciated by both patients with ALS and their families at different stages of the disease process, even when consultations were provided virtually (Zwicker et al., 2023). In the context of ALS nursing and a palliative approach, our results underscore the relevance of involving and supporting the whole family, particularly when encountering parents with minor children. By routinely applying a more proactive and comprehensive perspective—one that addresses the parental perspective through, for example, initiating conversations about the child’s situation, supporting family communication, and continuously following up throughout the course of the illness—care can be better adapted to the everyday challenges and needs of the entire family. This has implications for both nurses’ clinical practice and their education as well as interprofessional team practice and education, particularly in relation to providing support to families in these situations.

A limitation of the study is the missing information on the number of potential participants who chose not to participate. Exploring the meaning of parenthood to both ill parents and co-parents presents limitations, as well as strengths. Merging perspectives risks blurring unique experiences, so the ill parents’ and the co-parents’ interviews were initially analyzed separately. Ongoing discussions within the research group ensured fair comparison. Ultimately, combined perspectives offered a deeper, more nuanced understanding of the parenthood, highlighting changed family dynamics and the disease’s impact. Identifying both individual and shared needs is crucial for developing tailored support for the whole family. Given the underrepresentation of familial ALS in this study, future research focusing on this group is needed to gain a more comprehensive understanding of parenthood in the context of hereditary ALS and enable comparisons with other inherited life-threatening conditions.

## Conclusion

In conclusion, as parents struggled to navigate their illness-ridden and transformed parenthood, they seemed to have to handle many of their difficulties on their own. To promote the overall health of families affected by ALS, more proactive and tailored support from healthcare is necessary, including from a parental perspective. Support that strengthens parents’ SOC, while helping them find balance in their grieving process, may be crucial to their own well-being, but also their ability to maintain a steady parental presence. This reinforces the importance of early integration of palliative care to ensure that appropriate and timely support is provided throughout the care process. The results of this study could inform the development of such support within ALS teams, palliative care and nursing practice, which may also be applicable to families facing other life-threatening, rapidly progressive conditions, including neurodegenerative diseases or terminal cancers.

## Supplemental Material

sj-docx-1-gqn-10.1177_23333936251348143 – Supplemental material for Transformed Parenthood in the Face of ALS: A Profound Struggle for Both Ill Parents and Co-parents Supplemental material, sj-docx-1-gqn-10.1177_23333936251348143 for Transformed Parenthood in the Face of ALS: A Profound Struggle for Both Ill Parents and Co-parents by Nina Malmström, Joakim Öhlén, Stefan Nilsson, Ingela Nygren, Peter M. Andersen, Birgitta Jakobsson Larsson and Anneli Ozanne in Global Qualitative Nursing Research
