# Supplementary material for: Transformed Parenthood in the Face of ALS: A Profound Struggle for Both Ill Parents and Co-parents
Source: Glob Qual Nurs Res. 2025 Jul 12;12:23333936251348143. doi: 10.1177/23333936251348143 (PMC12256732; doi:10.1177/23333936251348143)
Supplement: sj-docx-1-gqn-10.1177_23333936251348143 – Supplemental material for Transformed Parenthood in the Face of ALS: A Profound Struggle for Both Ill Parents and Co-parents [file sj-docx-1-gqn-10.1177_23333936251348143.docx]

# **Supplementary File 1. Main interview questions**

**Main interview questions on the meaning of parenthood used to guide the interviews:**

- What did parenthood mean to you at the beginning of the illness, and how has it changed over time?
- How do you view your parenthood now, and what does it mean to you?
- Has your perception of yourself as a parent changed during the course of the illness, and if so, how?
- How do you experience being a parent, based on the fact that you are ill/the other parent is ill?
- Can you describe whether you and the other parent agree or have different views on parenthood, your roles, what information the children should receive etc.?

**Follow-up questions:**

- Can you tell me more?
- What does that mean to you?
- How did you experience that?
- Would you like to add anything more from your perspective as a parent?

**Additional questions in cases of hereditary ALS:**

- You know that you/the other parent has a hereditary form of ALS. What are your thoughts about that?
- Do you talk about this at home? If so, what do you talk about? If not, why do you think that is – and would you like to talk about it?
